# Comparative Analysis of Complete Chloroplast Genomes of *Caltha scaposa* for Identification and Phylogenetic Analysis

**DOI:** 10.1002/ece3.73728

**Published:** 2026-05-27

**Authors:** Le Chen, Yongming Fan, Qihang Chen, Xiaonan Yu, Tai Gao

**Affiliations:** ^1^ Beijing Botanical Garden Management Office Beijing Floriculture Engineering Technology Research Center, Key Laboratory of National Forestry and Grassland Administration on Plant Ex Situ Conservation Beijing China; ^2^ College of Landscape Architecture Beijing Forestry University Beijing China; ^3^ School of Human Settlements North China University of Water Resources and Electric Power Zhengzhou China; ^4^ GMU‐GIBH Joint School of Life Sciences, The Guangdong‐Hong Kong‐Macao Joint Laboratory for Cell Fate Regulation and Diseases Guangzhou Medical University Guangzhou China

## Abstract

The complete chloroplast genome of *Caltha scaposa*, a high‐altitude wetland species native to the Himalayas and Hengduan Mountains, was assembled and characterized to clarify its phylogenetic position and explore plastome structural variation within the tribe Anemoneae. The circular genome is 160,219,155,293 bp in length and exhibits a typical quadripartite structure, including a LSC (large single copy) region of 79,994 bp, an SSC (small single copy) region of 17,075 bp, and a pair of IRs (inverted repeats) of 26,410 bp each. The genome encodes 132 genes, comprising 87 protein‐coding genes, 37 tRNA genes, and four rRNA genes. A total of 54 SSRs (simple sequence repeats) were identified, predominantly mononucleotide A/T repeats. Comparative analysis with nine related species from the genera *Anemone* and *Pulsatilla* revealed significant variation in IR boundaries. *Caltha scaposa* possesses the shortest IR regions, while all examined *Pulsatilla* species exhibit expanded IRs, resulting in structural rearrangements. Nucleotide diversity analysis indicated that intergenic spacers, especially *ndhE*‐*ndhG* (Pi = 0.1195), are highly variable, whereas tRNA genes are extremely conserved. Codon usage analysis revealed a bias toward A/T‐ending codons. Phylogenomic reconstruction based on 40 complete plastomes using maximum likelihood and Bayesian methods robustly placed *C. scaposa* as a sister lineage to the clade comprising *Anemone* and *Pulsatilla* within tribe Anemoneae. These findings clarify the systematic position of *C. scaposa* , highlight dynamic IR evolution in Anemoneae, and provide valuable genomic resources for future evolutionary and population genetic studies in Ranunculaceae.

## Graphical abstract

This study assembled and characterized the complete chloroplast genome of *Caltha scaposa*, revealing it has the shortest inverted repeat regions among examined Anemoneae taxa and identifying highly variable markers for future phylogenetic work. Phylogenomic analysis of 40 plastomes robustly resolves *C. scaposa* as the sister lineage to the Anemone–Pulsatilla clade, clarifying its systematic position within Ranunculaceae. These findings provide critical genomic resources for understanding plastome evolution and species diversification in high‐altitude wetland plants.

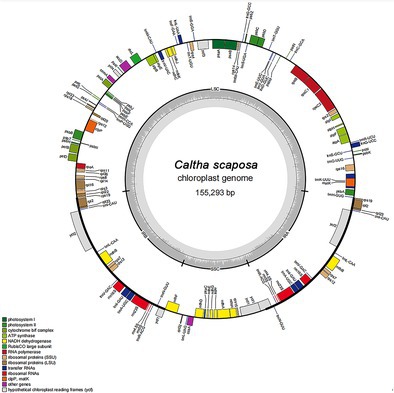

## 1 Introduction

The plants chloroplast genome is a highly conserved circular DNA molecule, typically ranging from 120 to 160 kb in size, and generally exhibits a quadripartite structure comprising two inverted repeats (IRs) that separate the large and small single‐copy regions (Shaw et al. 2007). This structural conservation, together with moderate sequence variation and maternal inheritance in most angiosperms, makes the plastome a valuable tool for phylogenetic reconstruction, species identification, and evolutionary investigations across various taxonomic scales (Wicke et al. 2011). In recent years, rapid advancement of high‐throughput sequencing has greatly facilitated plastome assembly, allowing for comparative genomic studies across diverse plant lineages and revealing both conserved features and lineage‐specific structural rearrangements (Daniell et al. 2021).

**FIGURE 1:**
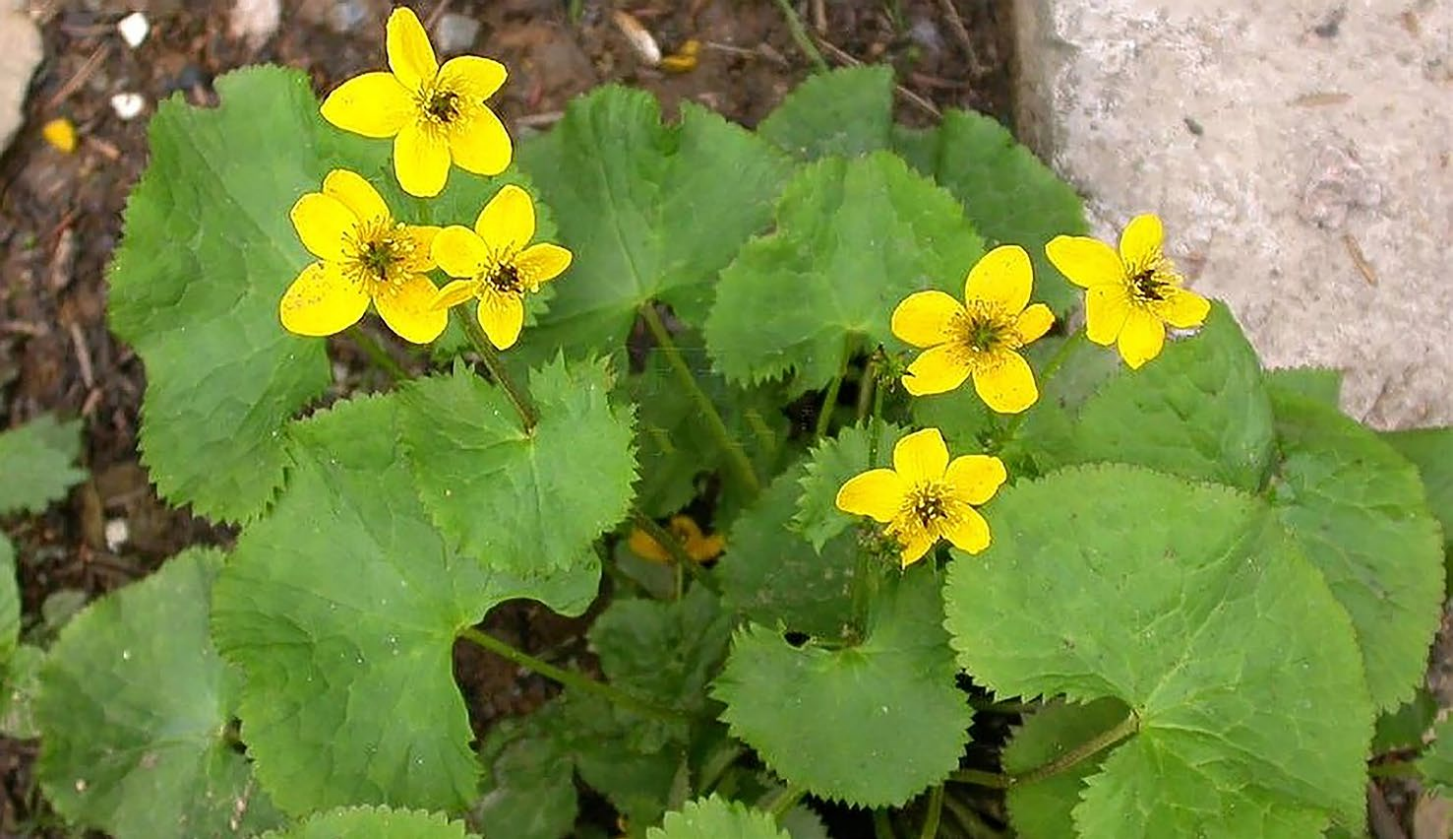
*Caltha scapose* (The photos are sourced from iPlant).

*Caltha* is a small but widely distributed genus within the family Ranunculaceae (Wang et al. 2001). *Caltha scapose* (Figure 1), a species native to the high‐altitude wetlands of the Himalayas and the Hengduan Mountains, represents an ecologically distinctive member adapted to cold and moist environments (Zhang, Meng, et al. 2017). Although nuclear and morphological data have provided some insights into the systematics of *Caltha* (Zhang et al. 2017), its precise phylogenetic placement within Ranunculaceae, particularly its relationship with other early‐diverging genera such as *Anemone* and *Pulsatilla*, remains incompletely resolved due to limited molecular evidence.

Recent phylogenomic studies have increasingly incorporated complete plastomes to resolve both deep and shallow nodes within Ranunculaceae (Ji et al. 2023). Notably, members of the tribe Anemoneae, including *Anemone*, *Pulsatilla*, and allied genera, have been shown to exhibit dynamic plastome evolution, characterized by IR boundary shifts, gene losses, and inversions (Liu et al. 2018). These structural changes often coincide with speciation events and may reflect adaptive responses to environmental pressures. Comparative analyses employing tools such as mVISTA and IR boundary mapping have proven effective in identifying both conserved coding regions and hypervariable non‐coding spacers, which serve as potential phylogenetic markers or indicators of genomic instability.

In this context, the plastome of *C. scaposa* offers a critical opportunity to expand our understanding of plastid genome evolution in basal eudicots by assembling and characterizing its complete chloroplast genome and conducting comparative analyses with closely related taxa, including eight *Pulsatilla* species.

## 2 Materials and Methods

### 2.1 Plant Materials and DNA Extraction

*Caltha scaposa* was collected from Deqin County, Diqing Tibetan Autonomous Prefecture, Yunnan Province, China. Upon collection, the leaves were immediately stored at 4°C. No specific permissions were required for the collection of plant samples from this location. Voucher specimens are deposited at the Aquatic Plant Application and Germplasm Enhancement Laboratory of the Yellow River Basin, North China University of Water Resources and Electric Power, NCWU. Genomic DNA was isolated from fresh leaf material employing the CretMag Multi Sample DNA Kit (Suzhou Cretaceous Biotechnology Co. Ltd., Suzhou, China), which utilizes a magnetic bead‐based extraction method as described by Zhou et al. (2022). The extracted DNA was evaluated for quality and quantity: the A260/A280 ratio was 1.78 as assessed by NanoDrop spectrophotometry, the concentration was measured at 83.2 ng/μL via Qubit fluorometry, and agarose gel electrophoresis revealed distinct bands with no evidence of degradation.

### 2.2 Sequencing, Complete Genomes Assembly, and Annotation

Sequencing was performed on the Illumina HiSeq 4000 platform (Shanghai OE Biotech. Co. Ltd., Shanghai, China) with 150 bp paired‐end reads. FastQC v0.12.0 (http://www.bioinformatics.babraham.ac.uk/projects/fastqc/, accessed: December 1, 2025) was used for quality control of the raw data (Chen et al. 2018). The complete chloroplast genome was assembled using GetOrganelle v1.7.7.1 (https://github.com/Kinggerm/GetOrganelle, accessed December 21, 2025) (Jin et al. 2020), with the cp genome of *Caltha palustris* (NCBI accession: MG581742) serving as a reference. The results showed that the assembled chloroplast genome achieved an average coverage depth of 150X. Annotation of the *C. scaposa* chloroplast genome was performed using the online platform CPGAVAS2 (http://www.herbalgenomics.org/cpgavas/) (Shi et al. 2019), followed by manual curation using Geneious R11 (Biomatters Ltd., Auckland, New Zealand). The circular genome map was generated using OGDRAW v1.2 (http://ogdraw.mpimp‐golm.mpg.de/, accessed January 24, 2026) (Lohse et al. 2007).

### 2.3 Identification of Simple Sequence Repeats

Simple sequence repeats (SSRs) were detected using MISA (https://webblast.ipk‐gatersleben.de/misa/, accessed December 22, 2025) (Beier et al. 2017). The minimum number of repeats for SSR motifs of lengths 1 to 6 was set to 10, 6, 5, 5, 5, and 5, respectively.

### 2.4 Comparative Analysis of cp Genomes

Comparative analyses of chloroplast genomes, with *Caltha scaposa* as the reference against eight closely related species, were performed using mVISTA in Shuffle‐LAGAN mode (https://genome.lbl.gov/vista/mvista/submit.shtml, accessed December 23, 2025) (Frazer et al. 2004). The boundaries of the inverted repeat (IR) regions were visualized using IRscope (https://irscope.shinyapps.io/irapp/, accessed: December 24, 2025) (Amiryousefi et al. 2018), comparing *C. scaposa* with nine related taxa. Using CPStools v2.5, nucleotide diversity (Pi) was calculated (Huang et al. 2024). Based on previous phylogenetic studies and taxonomic considerations, nine species were selected for comparative analyses. Their chloroplast genome sequences were retrieved from the NCBI database, with the following accession numbers: *Anemone taipaiensis* (NC_050873.1), *Anemone tomentosa* (NC_039451.1), *Pulsatilla campanella* (NC_061038.1), *Pulsatilla cernua* (MK860687.1), *Pulsatilla chinensis* (MK569491.1), *Pulsatilla dahurica* (MK860685.1), *Pulsatilla saxatilis* (NC_070435.1), and *Pulsatilla tongkangensis* (NC_061398.1).

### 2.5 Codon Usage Analysis

Codon usage bias was assessed using CodonW v1.4.2 (https://sourceforge.net/projects/codonw/files/codonw/Win32‐Executables‐1.4.2/, accessed: December 252025), from which relative synonymous codon usage (RSCU) values were calculated (Sharp and Li 1987). Following standard criteria, an RSCU value of 1.0 indicates no bias in codon usage, whereas values exceeding 1.0 reflect a preference for particular codons.

### 2.6 Phylogenetic Analysis

Phylogenetic analysis was performed on 40 complete chloroplast genomes, which included the newly sequenced *C. scaposa* genome from this study and 39 cp genome sequences downloaded from the NCBI database. *Paeonia obovata* (accession number JQ952561.1) was designated as the outgroup. For the analysis, protein‐coding sequences (CDS) were extracted using PhyloSuite v1.2.3, then aligned with MAFFT v7.505 (Katoh et al. 2019). Poorly aligned regions and divergent sites were removed with trimAl v1.2 (Capella‐Gutiérrez et al. 2009), and protein‐coding genes were refined using MACSE v2.0.3 (Ranwez et al. 2018). A phylogenetic tree was reconstructed employing both maximum likelihood (ML) and Bayesian inference methods, with ML bootstrap support evaluated using IQ‐TREE based on 5000 replicates (Katoh et al. 2019; Nguyen et al. 2015).

## 3 Results

### 3.1 Cp Genome Features of *C. scaposa*

**FIGURE 2:**
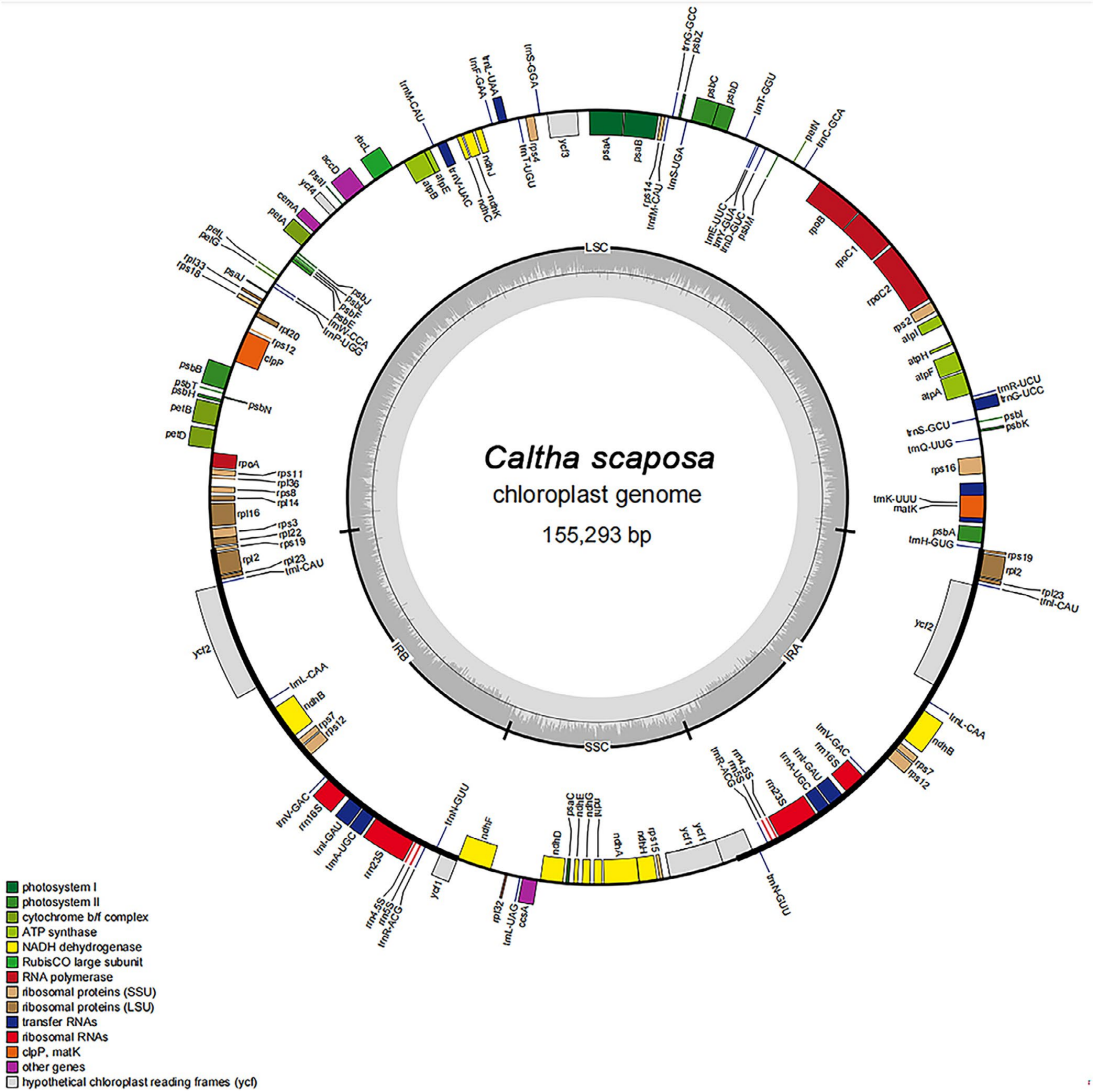
The circular complete cp genome map of *Caltha scaposa* . Genes shown outside the outer circle are transcribed clockwise, whereas those shown inside are transcribed counterclockwise.

The chloroplast genome of *C. scaposa* contains a total of 132 genes (by copy number), including 87 protein‐coding genes, 37 tRNA genes, and 4 rRNA genes (Figure 2). These genes are classified into three functional categories: photosynthesis‐related, self‐replication‐related, and others (Table 1). Due to the quadripartite structure, 17 genes are duplicated in the inverted repeat (IRs) regions. These include several protein‐coding genes including *rpl2*, *rpl23*, *rps7*, *rps19*, *ycf2*, 8 rRNA gene copies, and multiple tRNA genes (*trnI‐CAU*, *trnA‐UGC*). Owing to copy‐dependent repair via gene conversion between the two IR regions, the sequences of these duplicated genes are maintained with perfect identity. A total of 18 intron‐containing genes were identified in the *C. scaposa* chloroplast genome, including 15 genes (*ndhB*, *ndhA*, *petB*, *petD*, *atpF*, *rpl16*, *rpl2*, *rps16*, *rpoC1*, *trnK‐UUU*, *trnG‐UCC*, *trnL‐UAA*, *trnV‐UAC*, *trnI‐GAU*, and *trnA‐UGC*) with a single intron, and 3 genes (rps12, clpP, and ycf3) with two introns each, accounting for a total of 21 introns. Notably, the rps12 gene undergoes trans‐splicing: its 5′ exon is located in the large single‐copy (LSC) region, whereas the 3′ exon resides in the IR region (Figure 2). The complete cp genome sequence of *C. scaposa* has been deposited in GenBank under accession number PZ091414.

**TABLE 1:** The basic cp. genomes information of *Caltha scaposa* .

Category	Gene group	Gene name
Photosynthesis	Subunits of photosystem I	psaB, psaA, psaI, psaJ, psaC
	Subunits of photosystem II	psbA, psbK, psbI, psbM, psbD, psbC, psbZ, psbJ, psbL, psbF, psbE, psbB, psbT, psbN, psbH
	Subunits of NADH dehydrogenase	ndhJ, ndhK, ndhC, ndhB (2) #, ndhF, ndhD, ndhE, ndhG, ndhI, ndhA#, ndhH
	Subunits of cytochrome b/f complex	petN, petA, petL, petG, petB#, petD#
	Large subunit of rubisco	rbcL
	Subunits of ATP synthase	atpA, atpF, atpH, atpI, atpE, atpB
Self‐replication	Proteins of large ribosomal subunit	rpl33, rpl20, rpl36, rpl14, rpl16#, rpl22, rpl2(2) #, rpl23(2), rpl32
	Proteins of small ribosomal subunit	rps12(2) ##, rps16#, rps2, rps14, rps4, rps18, rps11, rps8, rps3, rps19(2), rps7(2), rps15
	Subunits of RNA polymerase	rpoC2, rpoC1#, rpoB, rpoA
	Ribosomal RNAs	rrn16S (2), rrn23S (2), rrn4.5S (2), rrn5S (2)
	Transfer RNAs	trnH‐GUG, trnK‐UUU#, trnQ‐UUG, trnS‐GCU, trnG‐UCC#, trnR‐UCU, trnC‐GCA, trnD‐GUC, trnY‐GUA, trnE‐UUC, trnT‐GGU, trnS‐UGA, trnG‐GCC, trnfM‐CAU, trnS‐GGA, trnT‐UGU, trnL‐UAA#, trnF‐GAA, trnV‐UAC#, trnM‐CAU, trnW‐CCA, trnP‐UGG, trnI‐CAU(2), trnL‐CAA(2), trnV‐GAC(2), trnI‐GAU(2)#, trnA‐UGC(2)#, trnR‐ACG(2), trnN‐GUU(2), trnL‐UAG
Other genes	Maturase	matK
	Protease	clpP##
	Envelope membrane protein	cemA
	Acetyl‐CoA carboxylase	accD
	c‐type cytochrome synthesis gene	ccsA
	Conserved open reading frames	ycf3##, ycf4, ycf2(2), ycf1(3)

*Note:* #, intron number; (n), gene copy number.

### 3.2 Analysis of SSRs

A total of 54 SSR loci were identified in the chloroplast genome of *C. scaposa* . Among them, 48 were mononucleotide repeats (A/T or C/G) and 6 were dinucleotide repeats (AT/AT). No tri‐ or higher‐order nucleotide repeats were detected. Within the mononucleotide repeats, A/T repeats were predominant, accounting for 47 loci, while only one C/G repeat was identified. The predominance of A/T repeats is consistent with the general characteristic of plant chloroplast genomes being A/T‐rich. The detailed distribution of all SSRs is shown in Table 2.

**TABLE 2:** Number of *Caltha scaposa* SSRs.

Repeats	5	6	7	8	9	10	11	12	13	14	Total
A/T	0	0	0	0	0	26	15	2	1	3	47
C/G	0	0	0	0	0	1					1
AT/AT	0	5		1							6

### 3.3 Comparative Analysis of Genome Structure in *C. scaposa* and Its Related Species

**FIGURE 3:**
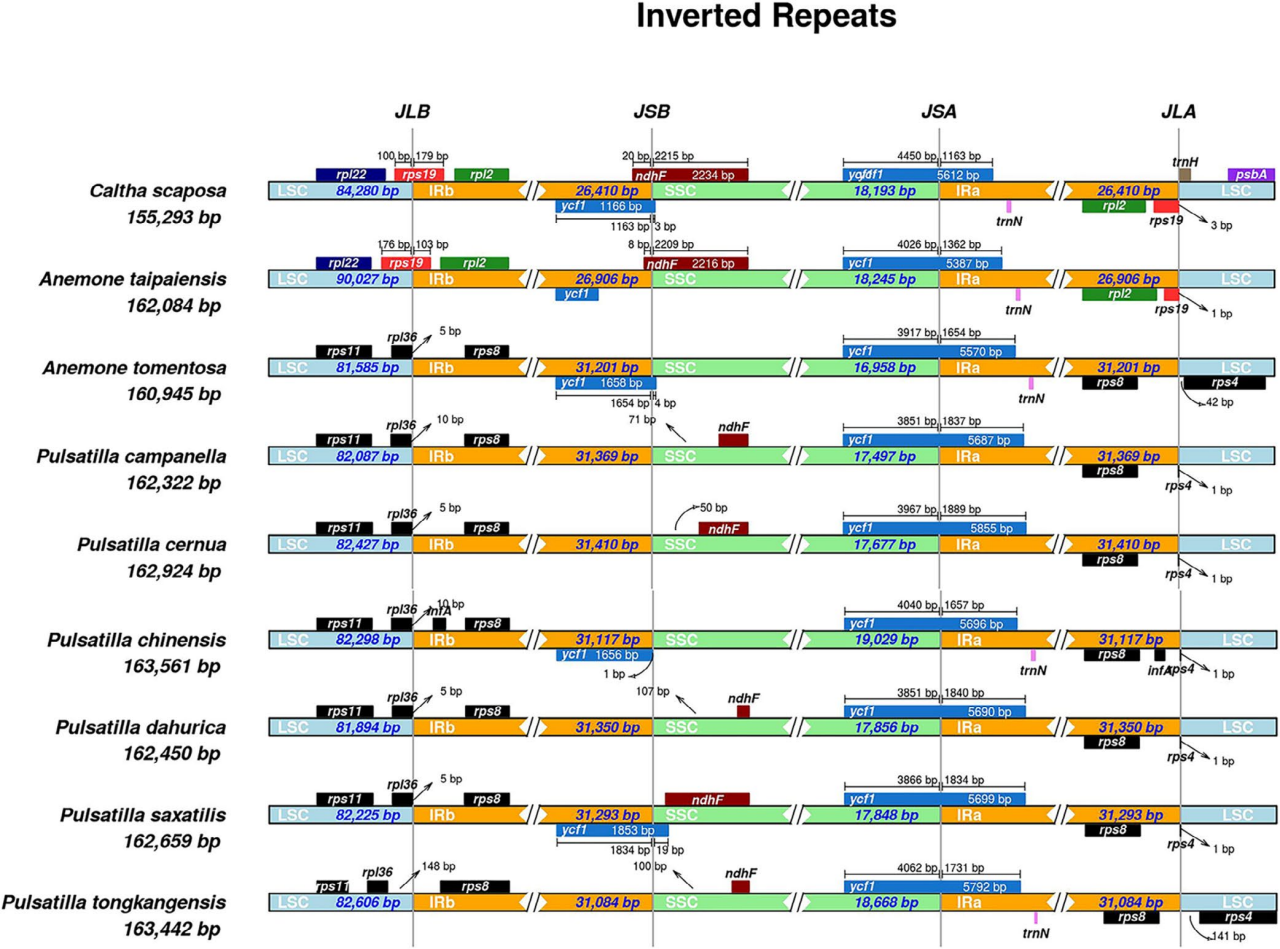
Comparison of the LSC, SSC, and IR regions among 9 selected cp genomes in the family *Caltha*, *Anemone*, and *Pulsatilla*. The gaps between the genes and the boundaries are indicated by the base lengths. Genes are denoted by colored boxes. JLB, junction between LSC and IRb; JSB, junction between IRb and SSC; JLA, junction between IRa and LSC; JSA, junction between SSC and IRa.

*Caltha scaposa* has the shortest IR regions, measuring 26,410 bp, whereas the two *Anemone* species have IR lengths of approximately 28,500 bp (Figure 2). In contrast, all *Pulsatilla* species possess significantly longer IRs, ranging from 31,117 to 31,293 bp. In both *Caltha* and *Anemone*, the rps19 gene is entirely located within the LSC region. However, in *Pulsatilla* species, the IRa region has expanded into the SSC region, resulting in truncation of the *ndhF* gene. The genes located at these boundaries include *rps4*, *rps19*, *rpl36*, *ndhF*, *ycf1*, and *trnH* genes. The position of the *trnH* gene also varies with the contraction or expansion of IRa, showing inconsistent distances from the IRa boundary across different species. The shifts in IR boundaries have directly altered the gene content in these junction regions. Notably, *rps19* and *ycf1* are the primary genes affected by these boundary movements. The *ycf1* gene spans the SSC/IRb junction and, in all examined species, appears as an incomplete pseudogene within IRb, while its intact copy resides in the SSC region. The length of the intact *ycf1* in the SSC varies slightly among species, reflecting the precise positions of the boundary shifts. The *ndhF* gene is located at the IRb/SSC boundary, with the larger part located in the SSC region. Due to asymmetric IR expansions, truncated pseudogene copies of *ndhF* and rps19 have formed in certain species (Figure 3).

**FIGURE 4:**
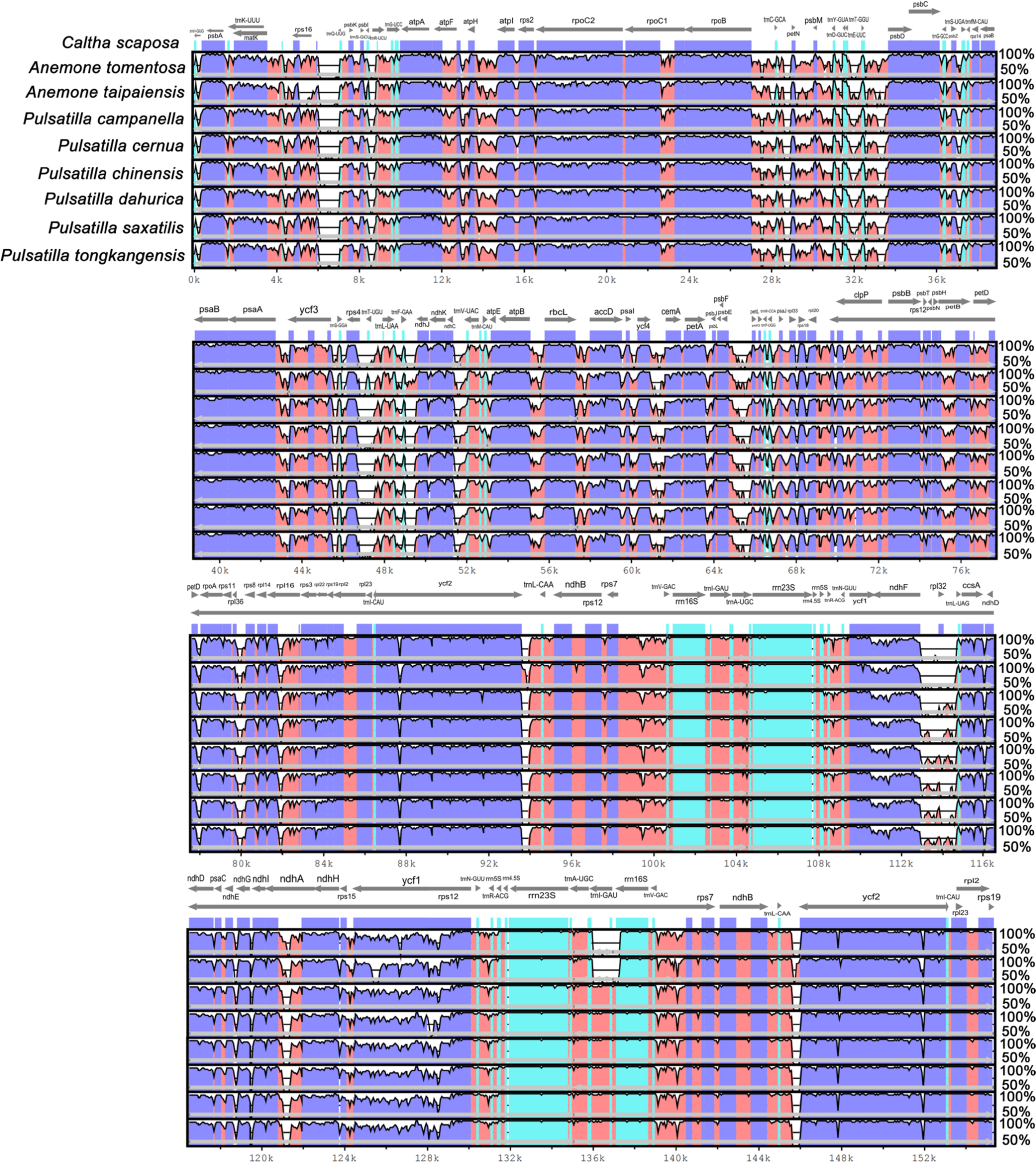
Sequence alignment of 9 *Anemone*, *Caltha*, *Pulsatilla* cp. genomes using the mVISTA program with *C. scaposa* as a reference. *x*‐axis: The coordinates in the cp genome, *y*‐axis: Percent identity within 50%–100%. The transcriptional direction of genes indicated by gray arrows. Regions where the identity curve falls to the baseline (~50%) indicate sequences that are highly divergent or absent in the query genome. The gene names are indicated directly above the dark gray arrows.

The mVISTA genome alignment plot illustrates the sequence conservation and variable regions among the chloroplast genomes of *C. scaposa* and its close relatives, using *C. scaposa* as the reference (Figure 4). The complete chloroplast genomes of nine Ranunculaceae species, including *C. scaposa* , two *Anemone* species, and seven *Pulsatilla* species—exhibit overall high sequence conservation, yet display several distinct non‐conserved regions concentrated in specific genomic intervals, accompanied by evident structural variations and elevated sequence divergence in intergenic spacers. Notably, in the genomic region spanning approximately 130–140 kb, which encompasses genes such as *ycf1*, *trnH‐GUG*, and *rps12*, all *Pulsatilla* species show markedly reduced sequence similarity compared to the reference, while *Caltha* and *Anemone* retain high conservation in this interval. This region corresponds precisely to the SSC, IRb junction, and its low conservation aligns closely with the previously observed IR expansion and boundary shifts, indicating that it is a hotspot for genomic rearrangements and sequence diversification. Additionally, in the intervals around 80–90 kb and 110–120 kb, *Pulsatilla* species exhibit multiple localized insertions, deletions, or repetitive segments, whereas *Caltha* and *Anemone* display continuous high conservation across the same regions. Furthermore, intergenic spacers generally show lower sequence conservation, with the *trnH‐psbA* and *trnL‐trnF* regions being particularly divergent. These non‐coding regions thus represent key sources of sequence variation among the studied taxa.

### 3.4 Codon Usage Patterns in *C. scaposa* and Its Close Relatives Cp Genomes

**FIGURE 5:**
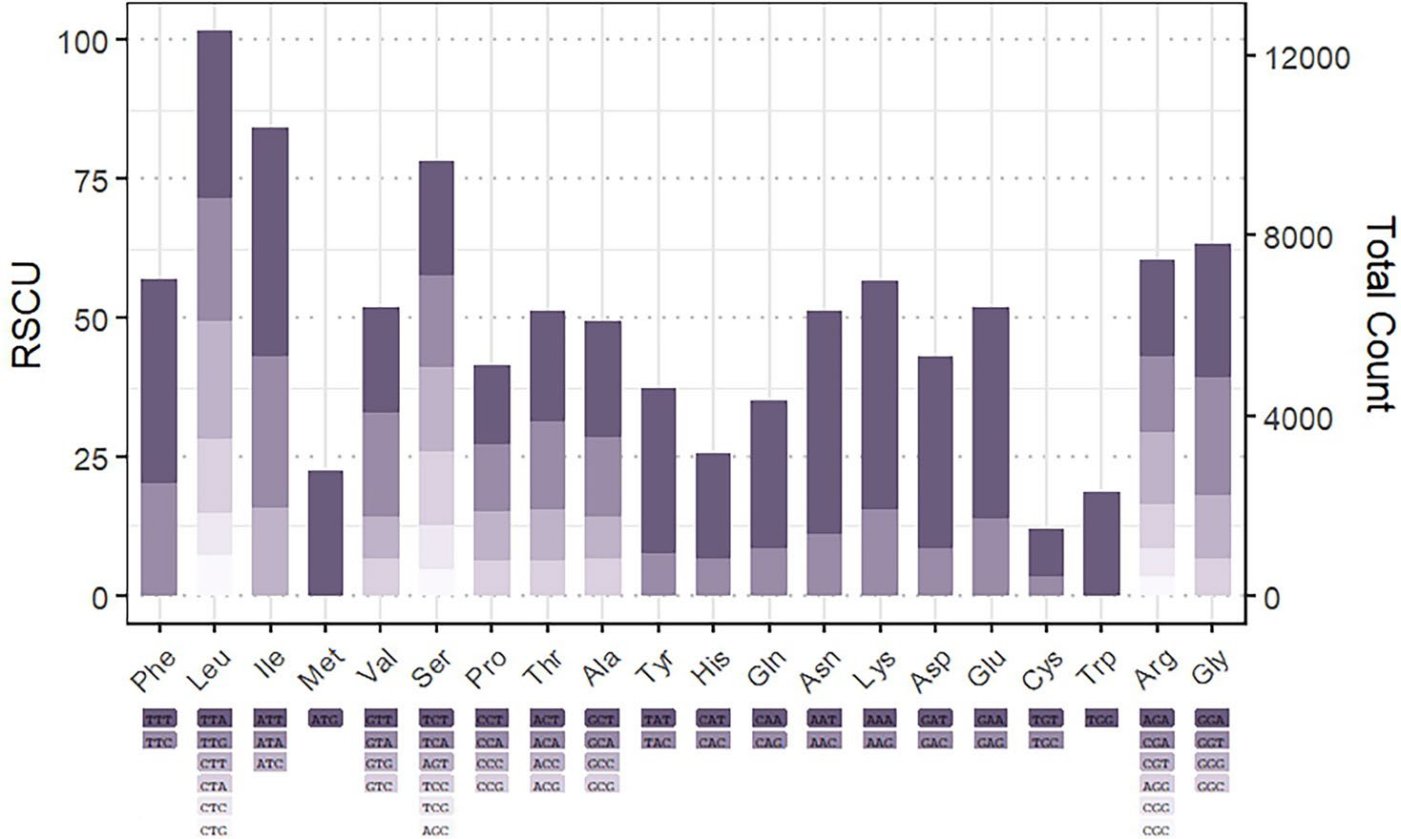
Codon usage in percentages (the right column) and RSCU values (the left column) of 20 amino acids. Each codon for an amino acid is shown with different colors.

Codon usage bias analysis of the *C. scaposa* chloroplast genome reveals significant differences in codon usage, indicating a pronounced codon preference (Figure 5). AAA (Lys) has the highest frequency at 41.234%, corresponding to 2171 occurrences. In contrast, AAG (Lys) is used much less frequently, at only 15.536%, suggesting that AAA is the predominant preferred codon for lysine. ATT (Ile) has a frequency of 41.139%, making it the most commonly used codon for isoleucine. GAA (Glu) shows a frequency of 38.024%, serving as the primary codon for glutamic acid. For arginine, AGA (17.322%) is the most frequently used, while CGC (3.305%) is the least used. For serine, TCT (20.664%) is the most frequent, whereas AGC (4.805%) is the least frequent. Most high‐frequency codons end with A or T, consistent with the general characteristic of plant chloroplast genomes favoring A/T‐ending codons. High‐frequency codons such as AAA, ATT, GAA, TTT, and AAT may represent optimal codons, potentially related to tRNA abundance or translation efficiency.

### 3.5 Nucleotide Diversity Analysis

**FIGURE 6:**
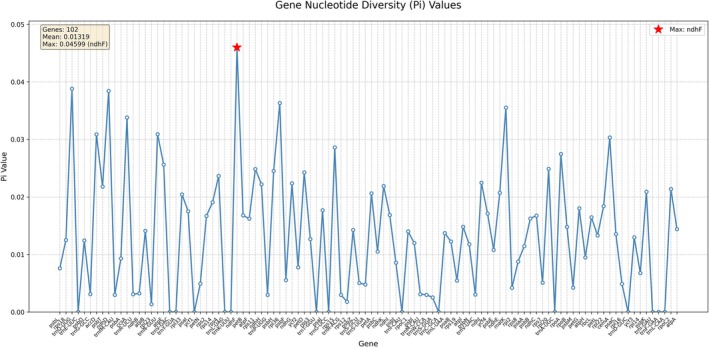
Sliding window analysis of cp genomes from 9 species. Nucleotide diversity (Pi) of intergenic regions in coding regions.

The Pi value range of the *C. scaposa* chloroplast genome is from 0.00000 to 0.119509(Figure 6). The region with the highest value is the intergenic spacer *ndhE‐ndhG*, with a value of 0.119509. A large number of regions show extremely low polymorphism, such as the tRNA genes *trnE‐UUC*, *trnV‐GAC*, and *trnY‐GUA*. Among the analyzed protein‐coding and RNA genes, *matK* (0.03554), *accD* (0.03089), and *ndhF* (0.045987) have relatively high Pi values, while *rpl23* (0.00138) and *rps12* (0.00179) exhibit extremely low Pi values. Almost all tRNA genes have a Pi value of 0.00000, indicating extreme sequence conservation. In general, intergenic regions show polymorphism values that are higher than or significantly higher than those in gene‐coding regions. Regions of high polymorphism are mainly concentrated in the intergenic spacers.

### 3.6 Phylogenetic Tree Construction and Analysis of Species Clusters

**FIGURE 7:**
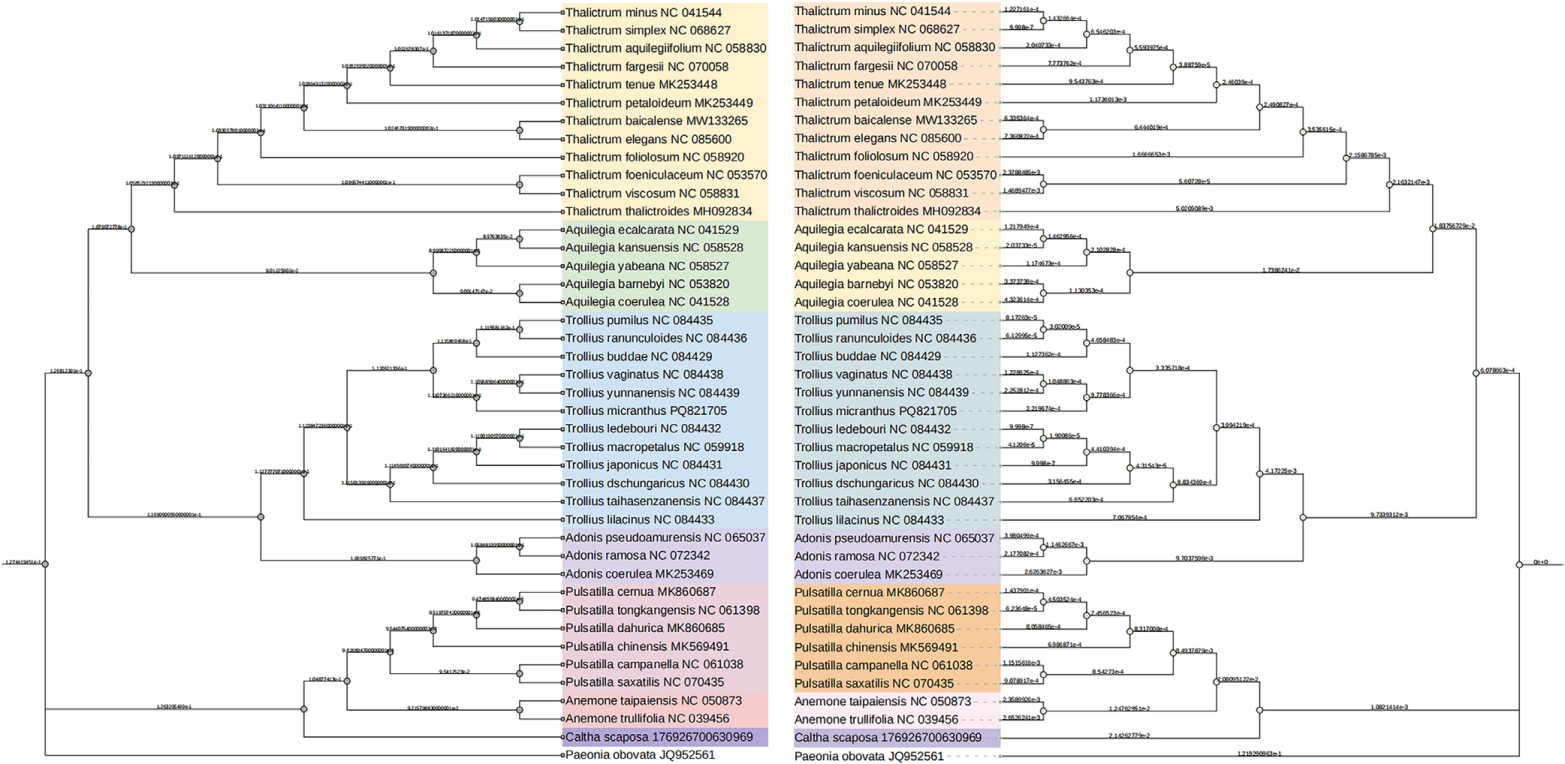
ML phylogenetic (left) and Bayesian tree (right) of 40 complete cp genomes. Bootstrap values and branch lengths are shown near each node. Different colors represent the differences in the clustering results.

Based on the CDS region of the chloroplast genome and using *Paeonia obovata* as the outgroup, phylogenetic trees constructed by maximum likelihood and Bayesian inference methods clearly reconstruct the classical phylogenetic relationships within the core subfamilies of Ranunculaceae (Figure 7). All analyses strongly support (bootstrap/posterior probability 100/100) the formation of a monophyletic clade within Ranunculaceae, consisting of the tribes Aquilegieae and Thalictreae as sister groups. Notably, the genus *Thalictrum* exhibits multiple well‐supported species‐level branches, reflecting a potentially rapid history of species diversification within this genus. This clade, in turn, forms a sister group to another monophyletic clade comprising the tribes Trollieae and Adonideae. The relationships among species within these tribes are clearly resolved, with generally high branch support values. This topology receives nearly complete support in both trees (maximum likelihood bootstrap 100, Bayesian posterior probability 100), which is fully consistent with recent studies based on genomic data and resolves long‐standing controversies regarding the relationships among these tribes.

*Caltha scaposa* is firmly nested within the tribe *Anemoneae*. It is not directly related to *Thalictreae* or *Trollieae* but is more closely related to the clade containing *Pulsatilla* and *Anemone*. *Caltha scaposa* is the sister group to the entire *Pulsatilla* and *Anemone* clade. This relationship receives 92% bootstrap support in the maximum likelihood tree and an even higher 93% posterior probability in the Bayesian tree. This strong supporting evidence indicates that after divergence from a common ancestor, one lineage evolved into the modern *Pulsatilla* and *Anemone*, while the other evolved into the *Caltha* lineage represented by *C. scaposa* (Odago et al. 2022; Raman et al. 2020). The divergence between *C. scaposa* and the most recent common ancestor of *Pulsatilla* and *Anemone* occurred earlier than the divergence between *Pulsatilla* and *Anemone* themselves. *Paeonia obovata*, used as the outgroup in the analysis, is consistently placed outside all core lineages of Ranunculaceae. It is extremely long branch length confirms the distant phylogenetic relationship and prolonged independent evolutionary history between *Paeonia* and the core Ranunculaceae lineages, while also highlighting the relatively closer relationships among the various groups within Ranunculaceae (Rui et al. 2025). The highly supported phylogenetic tree provides compelling molecular systematic evidence for the taxonomic delineation within Ranunculaceae, particularly among genera in the tribe Anemoneae. *C. scaposa* , as a clearly defined independent evolutionary branch, is positioned as a distinct lineage within the tribe Anemoneae of Ranunculaceae, forming a sister group relationship with the clade comprising *Pulsatilla* and *Anemone*. Its status at the genus level is fully confirmed. With the phylogenetic position of *C. scaposa* clarified, subsequent research can more precisely use this as a basis to conduct comparative genomics analyses, investigate species formation mechanisms, and estimate the divergence time between *Caltha* and the *Pulsatilla*‐*Anemone* lineage by integrating fossil evidence.

## 4 Discussion

As reported by Daniell et al. (2016), plant chloroplast genomes typically display a highly conserved quadripartite structure, comprising large single‐copy (LSC) and small single‐copy (SSC) regions separated by two inverted repeat (IR) regions. The complete chloroplast genome of *C. scaposa* provides crucial molecular evidence for resolving the phylogenetic position of the genus *Caltha* within the early‐diverging eudicot family Ranunculaceae. Our phylogenomic analyses, based on 40 complete plastomes, robustly place *C. scaposa* as the sister group to the clade comprising *Anemone* and *Pulsatilla*, with high bootstrap (92%) and posterior probability (93%) support. This finding strongly corroborates the monophyly of the tribe Anemoneae and clarifies that *Caltha* represents an independent evolutionary lineage that diverged prior to the split between *Anemone* and *Pulsatilla*. Although Liu et al. (2018) first proposed the sister relationship between *Caltha* and the (*Anemone* and *Pulsatilla*) clade, our study substantially advances this finding by employing 40 complete plastomes. Our results confirm that the *Caltha* lineage diverged prior to the split between *Anemone* and *Pulsatilla*.

Comparative structural analysis revealed significant variation in the inverted repeat (IR) boundaries among *Caltha*, *Anemone*, and *Pulsatilla*. Notably, *C. scaposa* possesses the shortest IR regions (26,410 bp), while all examined *Pulsatilla* species exhibit markedly expanded IRs (31,117 ~ 31,293 bp). This expansion in *Pulsatilla* has led to the truncation of the *ndhF* gene at the IRb/SSC junction, a structural rearrangement not observed in *Caltha* or *Anemone*. These lineage‐specific IR dynamics are consistent with a growing body of evidence in angiosperms showing that IR expansion/contraction is a major driver of plastome evolution (Hu et al. 2024). Specifically, our results go beyond Liu et al. (2018) by pinpointing IR expansion as the cause of *ndhF* truncation exclusively in *Pulsatilla*, which was not examined in their study. Furthermore, a recent study on the plastomes of the tribe Anemoneae (Hu et al. 2024) reported similar IR‐boundary shifts correlating with *ndhF* integrity, supporting our conclusion. Song et al. (2024) also found structural variation of the *ndhF* gene at IR boundaries in *Delphinium* and identified *ndhF*‐*trnL* and *ycf1* as hypervariable molecular markers for phylogenetic studies. These cross‐generic comparative analyses suggest that IR boundary dynamics may be a common mechanism of plastome evolution in Ranunculaceae and even basal eudicots. The mVISTA alignment further highlighted that the genomic region around 130–140 Kb (encompassing *ycf1*, *trnH*, and *rps12*) is a hotspot for sequence divergence, particularly in *Pulsatilla*, which directly correlates with these dynamic IR boundary shifts. These findings underscore that IR boundary dynamics are a major driver of structural and sequence evolution within the Anemoneae tribe.

Nucleotide diversity (Pi) analysis identified the intergenic spacer *ndhE‐ndhG* as the most variable region (Pi = 0.1195), while tRNA genes were found to be extremely conserved (Pi≈0.0000). This pattern, where non‐coding regions harbor higher polymorphism than coding regions, is typical of plant plastomes (Bian et al. 2024) and highlights the utility of these hypervariable spacers as potential molecular markers for population genetics and species identification within *Caltha* and its relatives. Compared to Liu et al. (2018), who did not perform nucleotide diversity scanning, our Pi analysis provides the first quantitative assessment of variable hotspots in the *Caltha* plastome, directly enabling marker development for this genus. Furthermore, the codon usage bias in *C. scaposa* , characterized by a strong preference for A/T‐ending codons, aligns with the overall A/T‐rich nature of its plastome and may reflect optimization for translational efficiency (Ji et al. 2023). To place this finding in a broader evolutionary context, we compared codon usage patterns between *Caltha* and the genera *Aconitum* and *Delphinium* using recently published Ranunculaceae plastome data. The A/T bias in *C. scaposa* is highly consistent with that of the congeneric species *C. palustris* , indicating that A/T preference is a general feature of Ranunculaceae plastomes, a conclusion further supported by previous studies on the tribe Anemoneae (Hu et al. 2024). Additionally, a study on *Ranunculus muricatus* (Wan et al. 2025) also reported a similar A/T bias pattern, further confirming the conservation of A/T preference in Ranunculaceae.

In conclusion, the plastome of *C. scaposa* not only resolves its long‐debated systematic position but also reveals lineage‐specific genomic features that contribute to our understanding of plastid genome evolution in basal eudicots. The clear sister relationship between *Caltha* and the (*Anemone* and *Pulsatilla*) clade (Szczecińska and Sawicki 2015), coupled with distinct IR structures and sequence divergence patterns, provides a solid foundation for future studies on the tempo and mode of diversification in the Anemoneae tribe.

## Author Contributions

**Le Chen:** conceptualization (equal), data curation (equal), formal analysis (equal), funding acquisition (equal), investigation (equal), methodology (equal), project administration (equal). **Yongming Fan:** conceptualization (equal), data curation (equal), formal analysis (equal), funding acquisition (equal), investigation (equal), methodology (equal), project administration (equal). **Qihang Chen:** conceptualization (equal), data curation (equal), formal analysis (equal), funding acquisition (equal), investigation (equal), methodology (equal), project administration (equal). **Xiaonan Yu:** conceptualization (equal), data curation (equal), formal analysis (equal), funding acquisition (equal), investigation (equal), methodology (equal), project administration (equal). **Tai Gao:** conceptualization (equal), data curation (equal), formal analysis (equal), funding acquisition (equal), investigation (equal), methodology (equal), project administration (equal).

## Funding

This research was funded by the China National Botanical Garden (Northern Garden), grant number T000002812929.

## Conflicts of Interest

The authors declare no conflicts of interest.

## Data Availability

The chloroplast genome sequence of *Caltha scaposa* has been submitted to NCBI (accession number: PZ091414).
